# mHealth for Schizophrenia: Patient Engagement With a Mobile Phone Intervention Following Hospital Discharge

**DOI:** 10.2196/mental.6348

**Published:** 2016-07-27

**Authors:** Dror Ben-Zeev, Emily A Scherer, Jennifer D Gottlieb, Armando J Rotondi, Mary F Brunette, Eric D Achtyes, Kim T Mueser, Susan Gingerich, Christopher J Brenner, Mark Begale, David C Mohr, Nina Schooler, Patricia Marcy, Delbert G Robinson, John M Kane

**Affiliations:** ^1^Dartmouth CollegeHanover, NHUnited States; ^2^Boston UniversityBoston, MAUnited States; ^3^University of PittsburghPittsburgh, PAUnited States; ^4^Mental Illness Research, Education and Clinical Center (MIRECC), Center for Health Equity Research (CHERP)Department of Veterans AffairsPittsburgh Health Care SystemPittsburgh, PAUnited States; ^5^Dartmouth-HitchcockLebanon, NHUnited States; ^6^Cherry Health and Division of Psychiatry and Behavioral Medicine, Michigan State UniversityGrand Rapids, MIUnited States; ^7^Independent ConsultantPhiladelphia, PAUnited States; ^8^Northwestern UniversityChicago, ILUnited States; ^9^State University of New York Downstate Medical CenterBrooklyn, NYUnited States; ^10^Northwell HealthGreat Neck, NYUnited States; ^11^Hofstra Northwell School of MedicineHempstead, NYUnited States

**Keywords:** mHealth, schizophrenia, technology, illness management, symptoms, relapse, engagement, adherence, smartphone

## Abstract

**Background:**

mHealth interventions that use mobile phones as instruments for illness management are gaining popularity. Research examining mobile phone‒based mHealth programs for people with psychosis has shown that these approaches are feasible, acceptable, and clinically promising. However, most mHealth initiatives involving people with schizophrenia have spanned periods ranging from a few days to several weeks and have typically involved participants who were clinically stable.

**Objective:**

Our aim was to evaluate the viability of extended mHealth interventions for people with schizophrenia-spectrum disorders following hospital discharge. Specifically, we set out to examine the following: (1) Can individuals be engaged with a mobile phone intervention program during this high-risk period?, (2) Are age, gender, racial background, or hospitalization history associated with their engagement or persistence in using a mobile phone intervention over time?, and (3) Does engagement differ by characteristics of the mHealth intervention itself (ie, pre-programmed vs on-demand functions)?

**Methods:**

We examined mHealth intervention use and demographic and clinical predictors of engagement in 342 individuals with schizophrenia-spectrum disorders who were given the FOCUS mobile phone intervention as part of a technology-assisted relapse prevention program during the 6-month high-risk period following hospitalization.

**Results:**

On average, participants engaged with FOCUS for 82% of the weeks they had the mobile phone. People who used FOCUS more often continued using it over longer periods: 44% used the intervention over 5-6 months, on average 4.3 days a week. Gender, race, age, and number of past psychiatric hospitalizations were associated with engagement. Females used FOCUS on average 0.4 more days a week than males. White participants engaged on average 0.7 days more a week than African-Americans and responded to prompts on 0.7 days more a week than Hispanic participants. Younger participants (age 18-29) had 0.4 fewer days of on-demand use a week than individuals who were 30-45 years old and 0.5 fewer days a week than older participants (age 46-60). Participants with fewer past hospitalizations (1-6) engaged on average 0.2 more days a week than those with seven or more. mHealth program functions were associated with engagement. Participants responded to prompts more often than they self-initiated on-demand tools, but both FOCUS functions were used regularly. Both types of intervention use declined over time (on-demand use had a steeper decline). Although mHealth use declined, the majority of individuals used both on-demand and system-prompted functions regularly throughout their participation. Therefore, neither function is extraneous.

**Conclusions:**

The findings demonstrated that individuals with schizophrenia-spectrum disorders can actively engage with a clinically supported mobile phone intervention for up to 6 months following hospital discharge. mHealth may be useful in reaching a clinical population that is typically difficult to engage during high-risk periods.

## Introduction

Patients, practitioners, and policy makers are increasingly enthusiastic about the use of mHealth approaches that can bring much needed resources (eg, information, assessment, and treatment) to people with chronic illnesses [1-3]. A recent meta-analysis of 12 studies conducted in the United States, Canada, United Kingdom, and India found that across countries the majority of people with schizophrenia-spectrum disorders own mobile phones, and many are interested in using them as tools to support the management of their illness [4]. Research examining mobile phone‒based mHealth programs for people with psychosis has shown that these approaches are feasible, acceptable, and clinically promising [5-10]. However, most mHealth initiatives involving people with schizophrenia have spanned periods ranging from a few days to several weeks and have typically involved participants who were clinically stable.

Schizophrenia has a prolonged and dynamic course typically consisting of periods of relative stability or remission interspersed with phases of symptomatic exacerbation that can last several months [11,12]. The months following hospital discharge are a particularly vulnerable period during which individuals are at increased risk for relapse and rehospitalization [13]. Whether people with schizophrenia are willing and able to engage in mHealth interventions during this high-risk period is unclear. On one hand, mHealth approaches are less constrained by clinic hours, location, or clinician availability and are therefore more flexible and accessible. On the other, mHealth programs require autonomous use and are limited in their personalization and adaptation capacities [14,15]. Consequently, patients might find mHealth interventions to be too effortful, formulaic, or repetitive and disengage.

With the current study, we set out to evaluate the viability of extended mHealth interventions for people with schizophrenia-spectrum disorders following hospital discharge. Specifically, we asked the following questions: (1) Can individuals be engaged with a mobile phone intervention program during this high-risk period?, (2) Are age, gender, racial background, and psychiatric hospitalization history associated with their engagement or persistence in using a mobile phone intervention over time?, and (3) Does engagement differ by characteristics of the mHealth intervention itself (ie, pre-programmed vs on-demand functions)? To address these questions, we examined mHealth intervention use and demographic and clinical predictors of engagement in 342 individuals who were given a mobile phone intervention for up to 6 months as part of a comprehensive technology-assisted relapse prevention program for people with psychosis following hospital discharge [16].

## Methods

### Procedures

The study was approved by the institutional review boards of the coordinating center, the participating sites, and the Committee for Protection of Human Subjects at Dartmouth College. Participants were drawn from a multisite Health Technology Program (HTP) implementation project that was conducted in partnership with 10 community mental health centers and outpatient clinics in eight US states between 2012 and 2015. HTP is described in detail elsewhere [16,17]. Briefly, the program offered individuals with psychotic disorders the opportunity to engage in a technology-assisted relapse prevention program for up to 6 months. As part of HTP, patients were offered an Android smartphone with the FOCUS [5,18] illness self-management program installed. Initially, a case manager introduced the mHealth intervention to patients and explained how to use the phone functions (eg, call, text, charge the battery) and FOCUS program (eg, respond to clinical assessment measures using the touchscreen, select on-demand tools). Once individuals demonstrated their proficiency, the case manager engaged them in a shared decision-making process to identify the three most relevant treatment targets from five possible FOCUS modules: medication adherence, mood regulation, sleep, social functioning, and coping with auditory hallucinations. Once treatment targets were selected, they were input into the mobile phone and patients could use FOCUS independently. Participants could call or meet with their case managers for technical support and troubleshooting. The FOCUS system prompted patients to engage in a brief assessment/intervention up to three times daily, focusing on their assigned treatment targets. In addition to pre-scheduled prompts, participants could access the treatment content for all five modules without restriction as part of the FOCUS on-demand functions. The mobile phone transmitted participant use data to a remote server regularly. Once data were uploaded, case managers at the different individual sites could view their assigned participants’ FOCUS activity via a secure online dashboard. HTP case managers met with participants for relapse prevention planning regularly, and data from the dashboard were available to inform these meetings. Brunette et al describe in detail the development of the Relapse Prevention Plan in which FOCUS was embedded [16].

### Participants

Individuals were eligible to participate in HTP if they were diagnosed with a psychotic disorder, were 18-60 years old, and had been discharged from psychiatric hospitalization within the past 60 days. The HTP sample consisted of 368 individuals. Four individuals were offered the FOCUS intervention but declined. Two individuals received a mobile phone but lost or sold it before the FOCUS program was activated and so did not generate mHealth use data. One individual was not offered FOCUS because his living environment did not permit the use of a mobile phone. Five individuals dropped out of HTP shortly after their baseline assessment and did not receive a mobile phone. Another 14 individuals provided fewer than 7 days of mobile phone data and were not included in the analyses because weekly engagement measures could not be calculated for them. Our final mHealth user sample consists of 342 individuals. These participants had a mean age of 35 years (SD 11). The sample was 62.3% (213/342) male, 50.0% (171/342) white, 25.1% (86/342) African-American, 10.8% (37/342) Hispanic, and 14.0% (48/342) were Asian, American Indian, Native Hawaiian, or more than one race. The majority (75.7%, 259/342)were single.

### Measures

Psychiatric diagnoses were based on medical records at the community mental health center or outpatient clinic where they received care and were confirmed by investigators at each site. Demographic information was collected during a baseline interview. Participants’ FOCUS use “events” were logged automatically by the mobile phone and transmitted to a study server when the device had connectivity. Four engagement outcomes were calculated for each individual: Days of mHealth Use represents the number of days a participant used any FOCUS function during the week. Days Responding to Prompts represents the number of days a participant responded to system-initiated prompts during the week. Days of On-Demand Use represents the number of days a participant initiated FOCUS use during the week. Average Daily On-Demand Use tallied how often within a day individuals self-initiated FOCUS functions. In initial descriptive tables, weekly engagement measures were summarized at the individual level for the entire time they participated in the study. Weekly measures were then characterized over time in longitudinal analyses.

### Overview of Analyses

The goal of longitudinal analyses was to estimate engagement over the course of the study. Participants, however, received the smartphone intervention for differing amounts of time and consequently had differing amounts of engagement data. Engagement data were missing for months without mobile phone data. Missing data may be a product of participants’ discontinuing FOCUS use prior to the end of the 6-month relapse prevention program or because they enrolled in HTP with less than 6 months left before the end of the project (these participants were informed that they may receive less than the full “dose” of the intervention when enrolling). For participants who dropped out prematurely, it is particularly important to take into account the relationship between available data and engagement. If a participant was less engaged with the mHealth intervention, they would be expected to be more likely to discontinue using it altogether. Therefore, modeling of engagement must assume missing data is informative on the engagement outcome values. Jointly modeling the longitudinal outcomes and the duration of available data allows an unbiased estimate of the association between predictors and the longitudinal outcome while appropriately accounting for missing data. In analyses including all participants (even those with less than 6 months of data), the results presented are from joint models. These models fit a longitudinal mixed-effects model for each engagement outcome simultaneously with a Cox proportional hazard model of duration of available data. Subgroup models among only those participants with 5-6 months of available data were performed via mixed-effects models. All longitudinal models included linear time terms, and quadratic time terms were retained if significant. To assess the effect of demographic factors and engagement, fixed effects were added to the time-trend models for each of the demographic factors of interest. Due to significant associations between age and gender (younger individuals were more likely to be male) and age and race (older individuals were more likely to be white), models for each demographic factor were fit separately. All longitudinal models include random individual-level intercept and slope terms to account for the correlation of outcomes over time within individuals.

## Results

The majority of participants were able to use the mobile phone and FOCUS program safely and without difficulty. One participant reported getting paranoid about the mobile phone and breaking it. Another participant reported only using it on “airplane mode” to avoid being tracked. Three participants deleted the FOCUS program from the phone. Another 21 participants reported their mobile phone lost or stolen over the course of their participation and requested a replacement device. One participant accidentally downloaded malware that rendered the phone inoperative and it needed to be replaced. At least two devices were pawned by participants.

Table 1 summarizes participant demographics and engagement rates. Participants included in this analysis had a study mobile phone for varying timeframes ranging from 8 to 183 days (mean 126 days, SD 52). Most participants (73.6%, 252/342) used the intervention for 3-6 months. On average, participants used the FOCUS program for 82% of the weeks they had the device. On average, participants had 9.5 (SD 7.4) meetings with HTP case managers in which they engaged in FOCUS-related topics (eg, device set-up and training, treatment target selection, technical troubleshooting, modification of prompting schedules, encouragement to use FOCUS strategies in the context of daily life).

**Table 1:** Individual demographic and engagement outcomes.

Demographic and study variables		Values
**Gender, n (%)**		
	Female	129 (37.7)
	Male	213 (62.3)
**Age, n (%)**		
	18-29	138 (40.3)
	30-45	135 (39.5)
	46-60	69 (20.2)
**Race, n (%)**		
	White	171 (50.0)
	African-American	86 (25.1)
	Hispanic	37 (10.8)
	Other (Asian, American Indian/Alaskan Native, Native Hawaiian, or more than one race)	48 (14.0)
**Marital status, n (%)**		
	Married	23 (6.7)
	Widowed/Divorced	57 (16.7)
	Single/Never married	259 (75.7)
**Months of mHealth use, n (%)**		
	<1	26 (7.6)
	1+	28 (8.2)
	2+	36 (10.5)
	3+	36 (10.5)
	4+	65 (19.0)
	5+	151 (44.2)
**Number of previous psychiatric hospitalizations, n (%)**		
	1-6	186 (55.2)
	7+	151 (44.8)
**Engagement measures, mean (SD)**		
	Days of mHealth Use per week	3.5 (1.9)
	Days Responding to Prompts per week	2.9 (2.0)
	Days of On-Demand Use per week	1.8 (1.4)
	Daily On-Demand Use	1.2 (1.8)
	Percentage of weeks used	82% (21%)
	Percentage of weeks responding to prompts	72% (28%)
	Percentage of weeks using on-demand functions	62% (28%)

Individuals who used FOCUS for 5-6 months of the relapse prevention program (44%) had higher average engagement throughout their participation than those who used FOCUS for less time. Their Days of mHealth Use were mean 4.3 (SD 1.8) per week; Days Responding to Prompts: mean 3.8 (SD 2.0) per week; Days of On-Demand Use: mean 1.9 (SD 1.5) per week; and Daily On-Demand Use: mean 1.3 (SD 1.8). In the Cox proportional hazard portion of the joint models, there was a significant association between level of engagement and likelihood of discontinuing use, with higher levels of engagement associated with lower risk of discontinuation. Greater number of psychiatric hospitalizations was also significantly associated with likelihood of discontinuing use, with a discontinuation hazard ratio of 1.4 (95% CI 1.1-1.8; *P*=.004) for 7+ hospitalizations compared to fewer hospitalizations.

**Figure 1:**
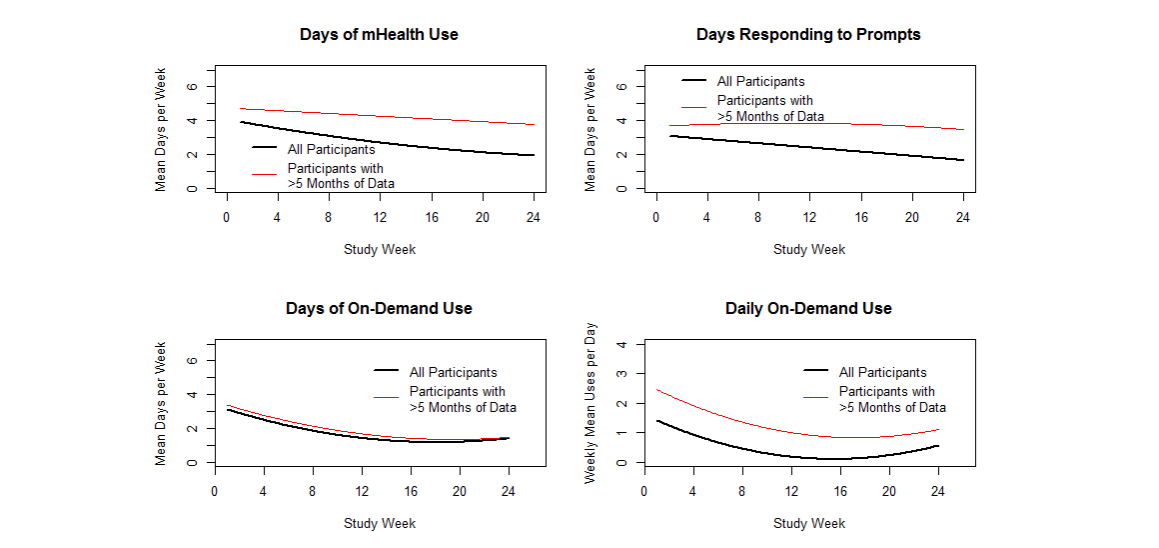
Model-based estimates of mHealth engagement over time.

The level of engagement with the mobile phone intervention declined over time (see Figure 1). The joint model results including all participants showed a curvilinear decline from an average of 3.9 uses in the first week to 1.9 uses in week 24. Days Responding to Prompts declined linearly and Days of On-Demand Use declined more steeply initially followed by a leveling off of use. Mean Days Responding to Prompts in the first week was 3.1 and in week 24 was 1.6. Mean Days of On-Demand Use was also 3.1 in the first week and 1.4 in week 24. Daily On-Demand Use also declined steeply initially followed by a leveling off in the later weeks (1.4 in week 1 and 0.6 week 24).

**Figure 2:**
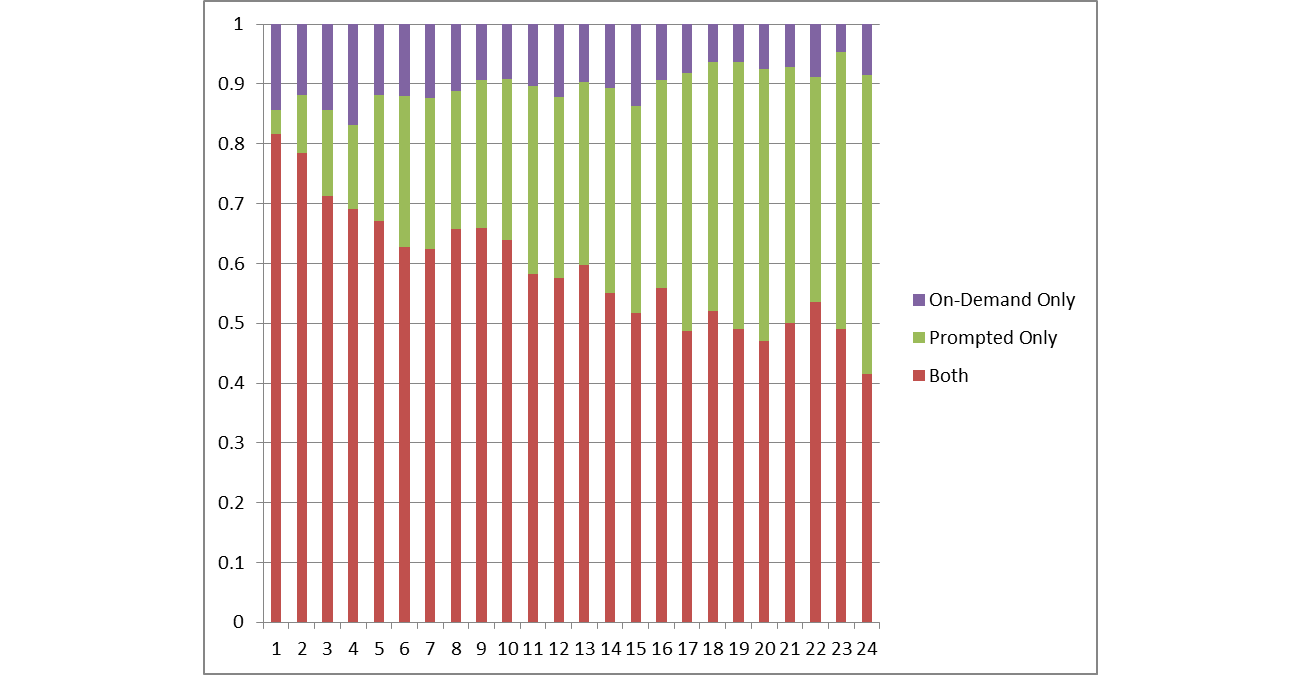
Proportion of engaged participants accessing on-demand features, prompted features, and both features, by study week.

If the analysis is restricted to only those participants who continued using FOCUS for 6 months, the declines in Days of mHealth Use and Days Responding to Prompts do not appear to be as steep. In this subset, engagement in week 24 remained high with a mean of 3.8 Days of mHealth Use, 3.5 Days Responding to Prompts, 1.5 Days of On-Demand Use, and 1.1 Daily On-Demand Use. Both prompted and on-demand features continued to be used throughout the study (see Figure 2).

Gender, race, age, and number of psychiatric hospitalizations were all found to be significantly associated with engagement outcomes (see Table 2). Females were significantly more engaged as measured by Days of mHealth Use, Days Responding to Prompts, and Daily On-Demand Use. On average, females used FOCUS on 0.42 days more per week than males and responded to prompts on 0.18 days more than males. On average, females also used on-demand features 1.61 times more per day than males. No significant association was seen between gender and Days of On-Demand Use.

White participants were the most engaged. They had significantly more Days of mHealth Use (0.69 more per week), Days Responding to Prompts (0.72 more per week), and Days of On-Demand Use (0.17 more per week) than African-American participants. White participants had significantly more Days Responding to Prompts (0.74 more per week) and Days of On-Demand Use (0.33 more per week) but less Daily On-Demand Use (1.32 less uses per day) than Hispanic participants.

Participants were categorized into three age groups: 18-29, 30-45, and 46-60. Participants aged 30-45 were significantly more engaged than younger participants (18-29 years) when considering Days of On-Demand Use (0.42 days more weekly) and Daily On-Demand Use (0.16 uses more per day). Older participants (46-60) were significantly more engaged in Days of On-Demand Use (0.48 days more weekly) and Daily On-Demand Use (1.78 uses more per day) when compared to those 18-29. However, they were significantly less engaged in Days Responding to Prompts (0.41 days fewer).

Participants with 7 or more psychiatric hospitalizations were significantly less engaged than those with fewer hospitalizations when considering Days of mHealth Use (0.2 days fewer per week), but no difference was seen in Days Responding to Prompts, Days of On-Demand Use, or Daily On-Demand Use.

**Table 2:** Joint longitudinal model results for engagement over time.

		Days of mHealth use		Days responding to prompts		Days of on-demand use		Daily on-demand use	
		Parameter estimate	*P*	Parameter estimate	*P*	Parameter estimate	*P*	Parameter estimate	*P*
Intercept		4.04 (0.10)	<.001	3.13 (0.072)	<.001	3.34 (0.085)	<.001	1.59 (0.11)	<.001
Study week		-0.14 (0.014)	<.001	-0.062 (0.007)	<.001	-0.24 (0.011)	<.001	-0.19 (0.014)	<.001
Study week 2		0.0021 (0.0006)	<.001	‒	‒	0.0067 (0.0004)	<.001	0.0063 (0.0005)	<.001
Male vs female		-0.42 (0.14)	<.01	-0.18 (0.076)	<.05	-0.11 (0.076)	.15	-1.61 (0.066)	<.001
**Race**									
	African-American vs white	-0.69 (0.13)	<.001	-0.72 (0.09)	<.001	-0.17 (0.082)	<.05	0.023 (0.068)	.73
	Hispanic vs white	-0.29 (0.23)	.22	-0.74 (0.14)	<.001	-0.33 (0.079)	<.001	1.32 (0.11)	<.001
	Other vs white	-0.46 (0.54)	.39	-0.95 (0.13)	<.001	-0.099 (0.094)	.29	-0.064 (0.12)	.58
**Age**									
	30-45 vs 18-29	0.34 (0.18)	.06	-0.10 (0.15)	.51	0.42 (0.076)	<.001	0.16 (0.067)	<.05
	46-60 vs 18-29	-0.27 (0.16)	.097	-0.41 (0.18)	<.05	0.48 (0.093)	<.001	1.78 (0.11)	<.001
**Previous hospitalizations**									
	1-6 vs 7+	0.21 (0.10)	<.05	-0.026 (0.084)	.76	-0.063 (0.076)	.41	-0.013 (0.066)	.84

## Discussion

### Principal Findings

To our knowledge, this study reports on the largest and longest implementation of an mHealth program for people with schizophrenia-spectrum disorders to date and is the first to systematically examine predictors of mobile phone intervention engagement among individuals who were recently discharged from a psychiatric hospitalization [19]. Our findings suggest that most participants (74%) were willing and able to use the FOCUS program successfully during this high-risk period for 3-6 months, which refutes the oft-stated concern that people with schizophrenia who are not clinically stable cannot engage in mHealth interventions successfully. On average, participants engaged with the mHealth program every other day. On days they engaged, they used on-demand (self-initiated) tools more than once a day.

Individuals’ continued use of the intervention over time was associated with their level of day-to-day engagement; people who engaged with the mHealth program more often used it for more months. This point is worth noting because active users undoubtedly encountered the same intervention content repeatedly; it is informative to see that more frequent program use did not lead to participant disengagement, but the contrary. Intervention program, patient, or provider characteristics may have contributed to these results; FOCUS intervention modules consist of brief skills training, practical exercises, and encouragement to use illness management techniques. Participants may have viewed FOCUS as a coaching or “booster” tool that had continued value even after their initial exposure to the content. Participants who were more engaged may have also had greater capacity to apply FOCUS strategies in their daily lives and reap the rewards, thus creating a reinforcing effect that would sustain their engagement. Finally, HTP case managers were charged with supporting their assigned participants’ use of the FOCUS intervention. There may have been some variability in how diligent they were in reviewing their patients’ mHealth use data via the dashboard and/or how active they were in encouraging daily and continuous use.

Several demographic and clinical variables were associated with engagement. Female participants were significantly more engaged and used the mHealth program on average one half-day more a week than males. White participants were more engaged and on average used the mHealth intervention almost one day more weekly than African-American participants and a third of a day more than Hispanic participants. Younger participants (age 18-29) were less engaged than older participants. Individuals with more severe psychopathology (as indicated by number of previous psychiatric hospitalizations) were less engaged than those with less severe psychopathology. Links between lower engagement in mental health treatment, male gender, minority background, younger age, and level of psychopathology have been found in the context of person-delivered care for people with schizophrenia [20]. Our data suggest that these patterns may apply to mHealth interventions as well. Despite having comparatively lower engagement, most younger participants, most male participants, most participants from minority backgrounds, and most participants with seven or more past hospitalizations typically used the FOCUS program multiple days a week over several months. While the mHealth intervention approach used in the study appears to be viable for these subgroups, attempting to optimize their engagement by developing adapted versions that can be tailored to subgroup needs may be warranted.

mHealth program functions were associated with engagement. Participants were exposed to FOCUS intervention content more often as a result of responding to system-initiated prompts than after initiating on-demand resources. Both types of intervention use declined over time (on-demand use had a steeper decline). This decline in use may be linked with patients feeling more capable of managing their illness and/or less likely to relapse (perhaps in part due to internalizing and practicing FOCUS self-management suggestions). Although mHealth use declined, the vast majority of individuals used both on-demand and system-prompted functions regularly throughout their participation, that is, neither function is extraneous. Thus, enabling both options in mHealth interventions for people with psychosis is recommended.

### Limitations

The study had several limitations. First, participants were provided with a fully functional mobile phone and data plan, which limits generalizability. While there was no contingent reinforcement or monetary incentive to use the FOCUS program, participants did gain access to other mobile phone resources (eg, Internet, games, texting) that might have indirectly influenced their FOCUS use. For example, a participant may have been more apt to notice and respond to FOCUS prompts if they took place when already using the phone to listen to music. We provided study participants with an Android smartphone to ensure the mHealth program worked reliably; the FOCUS system is a research tool that was not compatible with all commercial smartphone operating systems at the time of the study (eg, iOS, Windows). We also wanted to provide training, technical support, and troubleshooting solutions that would apply to all users. In the future, mHealth system that are compatible with a range of smartphone systems can be deployed, and clinical technology specialists who are embedded in health care systems may be able to provide technical and troubleshooting support to people using a wide array of devices [21]. Second, while we made every effort to document anomalies and unexpected events over the course of the study, there were likely technical (eg, prompting or data transmission failures, operating system updates that disabled the FOCUS program), and logistical barriers (eg, staff delays in replacing lost devices, delays in phones shipping to a study site) that went unidentified and unrecorded. In circumstances when these events hampered participants’ ability to use the mHealth intervention or resulted in unlogged use, engagement may have been underestimated. Finally, the power to make inferences about specific racial (especially Hispanic and “other”) or age groups is limited due to sample size and any relationships examined here should be confirmed in larger studies.

### Conclusions

As interest in mHealth for mental health treatment grows [22-25], it is important to evaluate which approaches are viable for different clinical populations (and when) and to gain a better understanding of the variables that may facilitate or hinder patient engagement with these novel interventions. This study provides evidence that individuals with schizophrenia-spectrum disorders can actively engage with a clinically supported mobile phone intervention for up to 6 months following hospital discharge; that gender, race, age, and history of psychiatric hospitalization were associated with their level of engagement; and that system-initiated mHealth functions led to proportionally more exposure to treatment content than on-demand tools, but that both were used regularly. Taken together, our findings indicate that the FOCUS mobile phone program may be a useful method to reach a clinical population that is typically difficult to engage in clinic-based services during high-risk periods. Future work should examine whether the use of FOCUS and other mHealth interventions can lead to clinically meaningful outcomes such as reduction in relapses.

## Abbreviations

HTP: Health Techology Program
